# A Novel CNN-Based Framework for Classification of Signal Quality and Sleep Position from a Capacitive ECG Measurement

**DOI:** 10.3390/s19071731

**Published:** 2019-04-11

**Authors:** Koshiro Kido, Toshiyo Tamura, Naoaki Ono, MD. Altaf-Ul-Amin, Masaki Sekine, Shigehiko Kanaya, Ming Huang

**Affiliations:** 1 Division of Information Science, Nara Institute of Science and Technology, Ikoma 630-0192, Japan; kido.koshiro.kb3@is.naist.jp (K.K.); nono@is.naist.jp (N.O.); amin-m@is.naist.jp (M.A.-U.-A.); skanaya@gtc.naist.jp (S.K.); 2 Future Robotics Organization, Waseda University, Tokorozawa 359-1192, Japan; t.tamura1949@gmail.com; 3 Department of Medical care Technology, Tsukuba International University, Tsuchiura 300-0051, Japan; m-sekine@tius.ac.jp

**Keywords:** capacitive coupling, electrocardiogram, CNN, deep learning, sleep positions

## Abstract

The further exploration of the capacitive ECG (cECG) is hindered by frequent fluctuations in signal quality from body movement and changes in sleep position. The processing framework must be fundamentally adapted to make full use of this signal. Therefore, we propose a new signal-processing framework that determines the signal quality for short signal segments (2 and 4 seconds) using a multi-class classification model (qua_model) based on a convolutional neural network (CNN). We built another independent deep CNN classifier (pos_model) to classify the sleep position. In the validation, 12 subjects were recruited for a 30-minute experiment, which required the subjects to lie on a bed in different sleeping positions. The short segments, classified as clear (C1 class) by the qua_model, were used to determine sleep positions with the pos_model. In 10-fold cross-validation, the qua_model for signals of 4-second length could recognize the signal of the C1 class at a 0.99 precision and a 0.99 recall; the pos_model could recognize the supine sleep position, the left, and right lateral sleep positions at a 0.99 averaged precision and a 0.99 averaged recall. Given the amount of data accumulated per night and the instability in the signal quality, this fully automatic processing framework is indispensable for a personal healthcare system. Therefore, this study could serve as an important step for cECG technique trying to explore the cECG for unconstrained heart monitoring.

## 1. Introduction

Cardiovascular diseases (CVDs) are still the number one cause of death globally according to the World Health Organization. Keeping the heart healthy helps prevent CVDs.

Long-term/daily monitoring could help prevent heart deterioration [1,2]. A bundle of wearable heart monitors using diverse physical principles has been devised, and they provide new ways to accumulate personal data/evidence. For example, ballistocardiography (BCG)—advantageous for its contactless measurement—records subtle vibrations in the human body generated by the mechanical activity of the heart pumping out arterial blood. Its use in heart rate monitoring has been established [3]. Another popular alternative is photoplethysmography (PPG), whose signal is generated by sensing changes in the concentration of hemoglobin in arterioles. Based on the PPG signal, the derivative time series of beat-to-beat interval is also used to estimate the physiological status of the heart [4].

In considering the fundamental depolarization and repolarization activities of the cardiac myocyte that causes the heart to contract and relax, measuring electrical signals is superior in minute and direct measurements of the heart. Electrical signals are measured by medical electrocardiogram (ECG), which is standard for monitoring the fundamental electrical activities of the heart and revealing diverse problems of the heart [5,6]. For this reason, recent years have actually witnessed a number of wearable devices that can measure the ECG signal being developed [1,7,8].

### 1.1. cECG and Prior Art on Modeling Based on cECG

Among these implementations, Ueno et al. proposed the capacitive sensing of the ECG signal (cECG) that can measure ECG signals without contact [8], and the cECG was further carried forward in heartrate-variability estimation [9]. Since cECG measures fundamental electrical signals, further uses are plausible with this technique, such as arrhythmia monitoring. However, its intrinsic properties are that (i) the signal quality abruptly changes due to body movement and (ii) the ECG vector that is projected onto the electrodes varies with different sleeping positions. These properties blur the perspective of cECG. To avoid the influence of the signal changes, an automatic processing procedure that distinguishes the noise caused by body movement is necessary. Similarly, to compensate for the variations in signals due to position changes, discrimination in the sleeping position is indispensable. Takano et al. recently devised a new system that simultaneously measures the cECG body proximity (BPx) and BCG signals and utilizes them to classify signals by the k-mean clustering method into the lateral and supine positions with an accuracy of 0.88 and 0.88, respectively. This study also verified the temporal accuracy of the cECG system and reported that the averaged standard deviation of the R-R interval (RRI) and the Q-T interval (QTI) were 1.08 and 3.78 ms, respectively [10]. However, the problems that hinder the full use of cECG signal—specifically, the strong fluctuations in signal quality due to body movement and changes of morphology due to changes in sleep position—have not been addressed. 

Multifarious methods have been proposed to assess the ECG signal. Behar et al. combined the moments and spectral information of the signal to decide its quality by support vector machine with a Gaussian kernel [11]. Orphanidou et al. used a decision tree to construct a binary classifier for the quality of wearable ECG signals [12]. These methods used the specific heartbeat waveform as matching templates; thus, segmentation and alignment of the ECG heartbeat were necessary. In view of the necessity in automatic signal quality classification so as to use the cECG better, Castro et al. used the linear discriminant analysis to classify the cECG signal into usable signal and noise. By using the ROC-optimized threshold, the binary classifier can reach an 0.85 averaged recall, 0.73 averaged precision, and 0.82 accuracy [13].

### 1.2. Contribution of this Study

Until now, most ECG signal classification methods have been based on handcrafted pre-defined features that may be sufficient for standard ECG signals but may not be competent for wearable/unconstrained measurements. With regard to this issue, a transition from the explicit feature definition to the automatic latent features extraction by algorithm would be necessary. A similar idea was implemented that used the convolutional neural network to classify arrhythmias using the MIT/BIH arrhythmia database [14].

Our aim was to devise a novel cECG-oriented signal preprocessing framework that successive waveform analysis such as R-R interval and QT interval detection and computation can be operated on. The input to the framework is the raw cECG data, and the hierarchical outputs are the signal quality annotations and the sleep position annotations without additional preprocessing such as the fiducial point detection. 

The convolutional neural network (CNN) technique has revived success in image processing and latent features generation [15,16]. The pooling manipulation of CNN endows itself with shift-invariant properties that are crucial in processing the cECG signal. The framework consists of two separated CNN models and is expected to classify the quality of the cECG signal into three categories (C1: Clear ECG signal, C2: Blurry ECG signal with clear R peaks, N: Noise) first, and then the sleep positions (S: Supine, L: left lateral, R: Right lateral) in sequence. To the best of our knowledge, our trial was the first to propose a fully automatic end-to-end preprocessing framework for cECG signals that can output a signal quality label and then a sleep position classification.

We intend to achieve fully automatic signal quality screening and position classification fundamentally different from previous studies based on two aspects: (1) Our framework does not require additional signal manipulation (i.e., R-peak detection and signal alignment). The signal is divided into short periods (e.g., 2 s or 4 s), and thrown into the framework directly. On the other hand, the framework should be adaptable to the abrupt random changes in signal quality; (2) this framework was constructed assuming that cECG will be applied to heart healthcare practically; therefore, a fully automatic end-to-end workflow is a prerequisite for the framework. This framework is meant to fill in the gap between sensor engineering and practical application to facilitate health informatics based on the cECG technique.

## 2. Materials and Methods

The cECG signal is specific to the implementation of the electrodes, and it is influenced by physiological differences among users. Therefore, we will briefly explain the cECG, then explain problems in practical use, and finally interpret the framework we constructed to tackle the aforementioned problems.

### 2.1. Capacitive ECG

**Figure 1:**
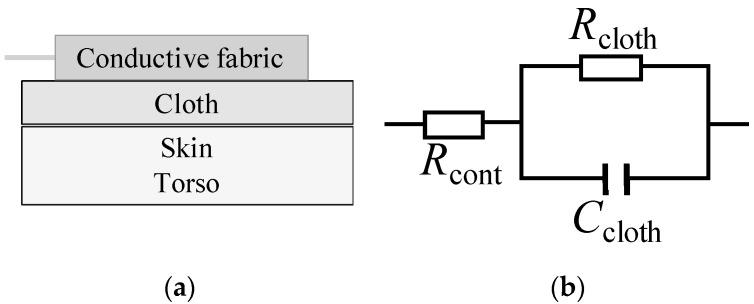
Illustration of the capacitive ECG (cECG) coupling (**a**) and the equivalent circuit of the capacitive coupling; (**b**) $R_{\mathrm{cont}}$ is the contact resistance between the cloth and the electrode, $R_{\mathrm{cloth}}$ is the resistance of the cloth, and $C_{\mathrm{cloth}}$ is the capacitance of the cloth.

The cECG is based on the capacitive coupling between the human body and an electrode, which uses the electrical insulating property of cloth to create a theoretically contactless ECG (Figure 1a). According to the equivalent circuit of the capacitive coupling (Figure 1b), the frequency-impedance response of the cECG can be derived as

(1) $$∥Z_{t}∥=R_{\mathrm{cont}}+\frac{R_{\mathrm{cloth}}}{\sqrt{{\left(2\pi R_{\mathrm{cloth}}C_{\mathrm{cloth}}\right)}^{2}+1}}$$

where $R_{\mathrm{cont}}$ is the contact resistance between the cloth and the electrode, $R_{\mathrm{cloth}}$ is the resistance of the cloth (150 MΩ), and $C_{\mathrm{cloth}}$ is the capacitance of the cloth (70 pF). Accordingly, the impedance $Z_{t}$ responds to the changes in the coupling area, the distance between the skin and the electrode (approximately the thickness or the cloth), the dielectric constant of the cloth, and signal frequencies. 

The cECG signal was collected by a cECG device (Cosmic ME, T14-2701) based on the design of Ueno et al. [8]. The electrodes of the device were made from a conductive fabric (2191FR, 0.003 MΩ/25 mm^2^, 3M^TM^, Austin, TX, USA). cECG signal extracted from the output pin of this device was converted to digital signal with an analog-to-digital converter (USB-6008, 12 bits in ±1V, National Instruments ^TM^, Austin, TX, USA) and collected by a LabVIEW (National Instruments ^TM^, Austin, TX, USA) program on a PC.

**Figure 2:**
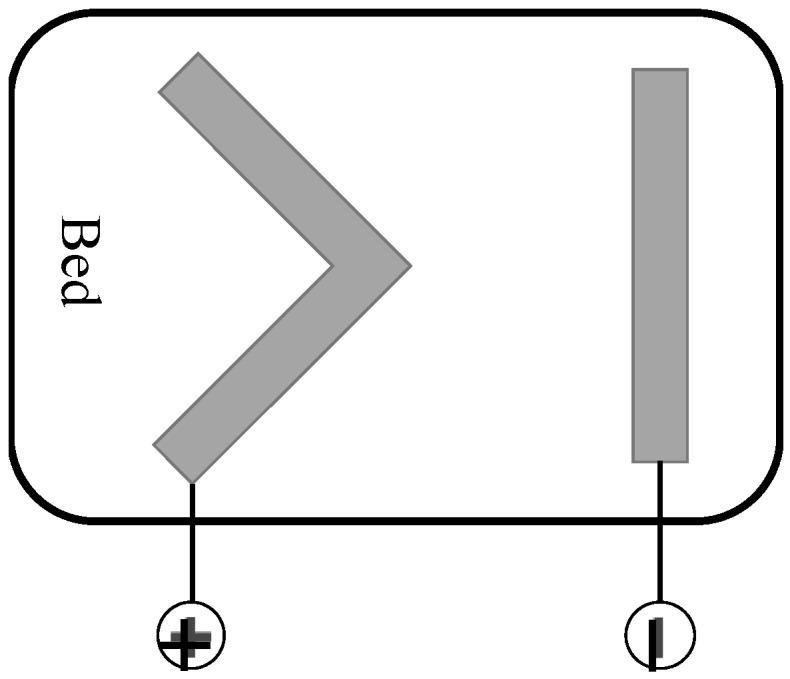
The configuration of the cECG on a bed. Two belt-shape electrodes were made from conductive fabric.

Figure 2 shows the configuration of the electrodes of the cECG. Both belt-shape electrodes are made from conductive fabric. The negative electrode is used to measure the electrical potential on the dorsum close to the heart. The positive electrode is positioned where its upper vertex is right under the hip. The positive electrode was designed in a V shape to properly measure the positive potential for different sleep positions. With this configuration, the system can measure the signal under different sleeping positions.

**Figure 3:**
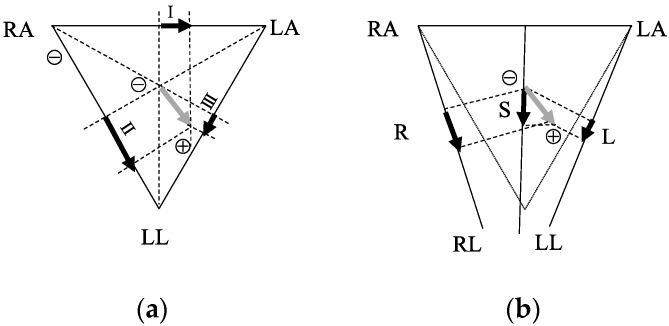
ECG vector projection onto the medical ECG limb leads (**a**) and the cECG. Grey arrows are the ECG vectors. Black arrows in (a) are the projections in leads I, II, and III; the black arrows in (**b**) are the projections of L, R, and S sleeping positions.

However, with this configuration, the projection of the ECG vector produced by the depolarization–repolarization cycle of the cardiomyocyte varied with different sleeping positions and this variation made the cECG signal difficult to interpret (Figure 3). By projecting the ECG vector into corresponding directions (Figure 3b), the theoretical inference is that the amplitude of the ECG waveform is minimal in the left lateral position, while the right lateral or supine positions would have clear waveforms. 

### 2.2. Data Collection and Organization

In our study, 12 subjects (males, age = 24.7 ± 2.8 years old) were recruited to collect the cECG data (sampling frequency: 300 Hz). The experiment protocol was as follows:

- Each subject was instructed to lie on a bed with cECG electrodes on top in a supine position (S) for the first 10 min, then a right lateral position (R) for 10 min, and finally the left lateral position (L) for 10 min.
- During the experiment, subjects were allowed to move their limbs without changing their position.

This series of experiments was approved by the Ethics Committee of the Nara Institute of Science and Technology. Following a detailed explanation of the investigation objective, the subjects gave their informed consent. 

To organize the raw data into a usable dataset, the following procedure was implemented:

1. Signal filtering with a band-pass filter (BPF) (Butterworth 2nd order, [0.05–40] Hz) for one record;
2. Signal quality annotation by a licensed clinical technician (ground truth of the qua_model) into C1: A clear ECG waveform, C2: A blurry ECG waveform with clear R peak, and N: A noise signal;
3. Automatic record segmentation with 50% overlapping (2 times lengths, i.e., 2 s and 4 s);
4. Sleep position annotation referring to experimental protocol (ground truth of the pos_model);
5. Data normalization for each short signal segment.

In the preprocessing stage, a band-pass FIR filter to remove the exogenous influences is standard for the medical ECG measurement [17]. For the unconstrained cECG, similar preprocessing was adopted [13].

Noteworthily, the signal preprocessing in step 1 is not adequate in removing noise from all sources for the unconstrained measurement of cECG. Influences such as limb/body movements may still exist. It is very difficult to remove all kinds of exogenous influences but to retain the spectrum with substantial physiological information ([0.05–40] Hz). In this continuous measurement environment with an unconstrained system, the amount of data should be sufficient, so we focused on extracting as much information as possible from the stable signal. By using the multi-class classification, we can extract as much information as possible from the stable signal (C1 class). Contrarily, we can discard the signal that is heavily contaminated by the influences (N class) or extract the physiological information partially, e.g., the time series of heart rate interval, from the signal that is influenced (N2 class).

In step 3, the dataset was generated with 50% overlapping. The data augmentation is a common measure to increase the diversity of the training data so as to mitigate the overfitting problem. 

For the modeling of sleep position, since the noise includes no information about sleep position and the whole ECG waveform is important in sleep position recognition, only the C1 signal will be used in sleep position classification.

### 2.3. CNN-Based Models and Framework Construction

**Figure 4:**
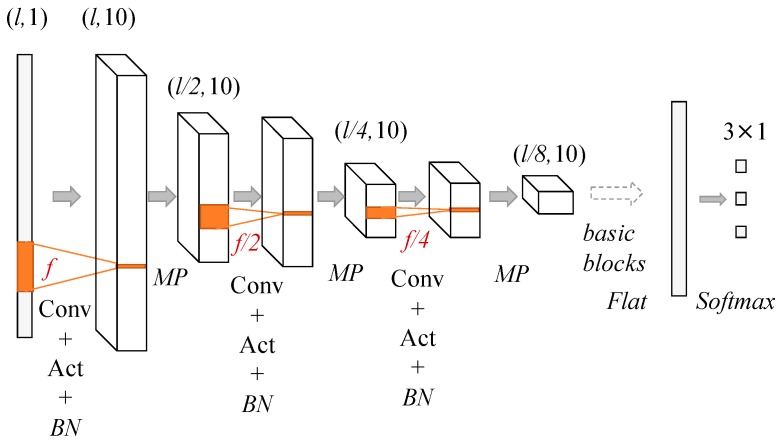
Convolutional neural network (CNN) structures used in building the qua_model and the pos_model. The dimensions of the input data decreased by 50% after each max pooling (MP) layer and the numbers of Conv layer are from 3 to 8.

We constructed CNN models, a qua_model for signal quality classification, and a pos_model for sleep position classification, with different numbers of convolutional layers to search for the best network structures. Generally speaking, a deeper neural network could extract more abstract features. However, the best depth depends on the specific problem. A model with excessive depth may hamper the learning process [18]. In this study, the problems that classify the signal quality and sleep position may not require as many as the convolutional layers in color image recognition. Therefore, in this study, we defined the structures of models with different numbers of convolutional layers ranging from 3 to 8. The general structure of the models can be seen in Figure 4. The depth of the models was decided by the number of the basic block that consists of the convolution (Conv) activation (Act) and Batch normalization (BN) layers. Since the max pooling layer (MP) was used after each basic block and the length of the filters (*l*) in the first Conv layer was 300 (1 s), the lengths of the filters in the following blocks were decreased by 50% from the previous Conv layer to keep the temporal resolution of the filters constant. Specifically, when the model with 8 Conv layers was used, the MP layer after the 7th basic block was removed, and the length of the filters in the 8th convolutional layer was the same as that in 7th Conv layer. Following the 8th basic block, the MP was applied. Since the numbers of the basic block were from 3 to 8, there were 6 CNN-based models to compare. The numbers of filters in all Conv layers were constant as 10. 

The qua_model and the pos_model were trained separately and the best structures of these two models would be different intrinsically. Therefore, the purpose of this experiment was to decide the best structures that can be characterized by the numbers of the basic blocks for the qua_model and the pos_model, respectively.

The two models were built based on 1D CNN, which can use the training data to adjust the weights of its filters to capture the latent features of the training data. The * symbol represents the convolutional operation and each filter will slide through the input with a constant stride (pre-set as 1 in this study),

(2) $$C_{p}^{r}=\sigma \left(I\times k_{p}^{r}+b_{p}^{r}\right).$$

Superscript *r* denotes the number of layers, and subscript *p* denotes the number of filters; *I* is the input to the convolution layer. As for the adjustable weights of a convolutional layer, *k* is the coefficients of the filters and *b* is the biases of the filters. 

Activation function σ( ) was then applied to the output of the filters. In this study, the rectified linear unit (ReLU) function

(3) $$\sigma \left(x\right)=\{\begin{array}{c} 1,\;x>0\\ 0,\;x\leq 0 \end{array}$$

was used for the convolution output.

In a deep neural structure, the output of a layer would be affected by its preceding layers and its internal covariance. In a complicated structure, the internal covariate shift (a phenomenon in which the differences between the distribution of output of an internal layer and the input) can be seen. This phenomenon may slow the learning process [19]. To mitigate this influence, the batch normalization (BN) technique was applied to the mini-batch data in the training process, by which the mean and standard deviation of this batch was calculated as $\mu_{B}$ and $\sigma_{B}^{2}$. Then, the data inside the mini batch were converted to

(4) $$\hat{x_{i}}=\gamma \frac{x_{i}-\mu_{B}}{\sqrt{\sigma_{B}^{2}+\epsilon }}+\beta$$

where γ and β were the new parameters for the model and ϵ was preset as 0.001. 

The MP was then applied to the output of the BN layer to reduce the dimensions and extract the shift-invariant features. After a couple of basic block and MP combinations, the output from the last MP will flatten and fully connect to the output layer (20% dropout between the flatten layer and the fully connect layer)

(5) $$O=\sigma^{o}\left(W\times f+b^{o}\right).$$

**Figure 5:**
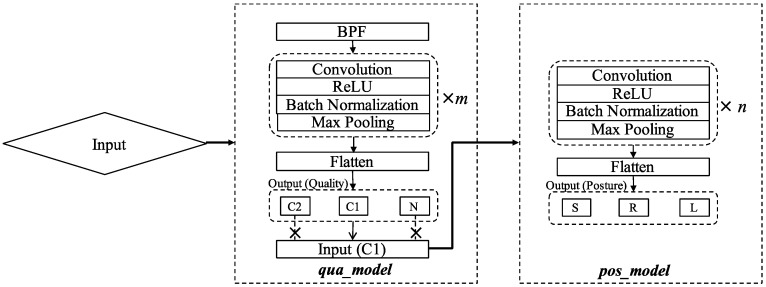
The workflow of the framework consisting of signal preprocessing and CNN-based classification. C1 samples, classified by the qua_model, were then fed to the pos_model for position classification. The italic *m* and *n* represent the best numbers of Conv layer in the two models.

The superscript o denotes the output layer; *O*, $O\in {ℜ}^{N}$, is the output of the network, and the superscript *N* is the number of classes in this study. On the right-hand side, the flattened vector *f* was fully-connected (denoted by the × symbol) to the output layer by adjustable weights *W* and $b^{o}$. Since both models were aimed at the multi-class classification problem, the $\sigma^{o}$ was the softmax function,

(6) σo(xi)=s(xi)=exi∑jNexj

where $x_{i}$ and $x_{j}$ are the outputs of neurons in the output layer. The structure of the CNN is illustrated by Figure 4 and the flow of the framework is shown in Figure 5. 

The signal quality changed abruptly due to body movement and position changes; therefore, the signal quality had to be checked frequently to avoid noise contamination that would degenerate the classification outputs. Hence, we defined the time length of the input signal to be 2 s and 4 s assuming that a longer time-length (in comparison with 1 s) would allow the models to extract more features. The dimensions of the input to the framework were (#sample, 600, 1) for 2 s and (#sample, 1200, 1) for 4 s; #sample is the number of training data, which will be interpreted later in model validation.

### 2.4. Validation

Tenfold cross-validation (CV), which is a standard method for model validation, was carried out to evaluate the qua_model and pos_model of different structures. In this way, 90% of the data were used in training, from which 10% of the training data were used to validate the learning process of each epoch; the other 10% of the data were separated out and used in the evaluation stage only. This process will be repeated for 10 times so that all the data in the dataset will be tested.

With the qua_model, multi-class classification (C1, C2, and N) was implemented. Similarly, with the pos_model, multi-class classification (S, L, and R) was implemented for the samples labeled as C1 by the qua_model. For the evaluation, the C1 and C2 are two major classes for evaluation of the qua_model. In the pos_model, all three positions are equally important and the position-specific evaluation by precision and recall were used as the criteria. The definitions of precision and recall are as follows:

(7) $$\mathrm{Precision}=\frac{\mathrm{TP}}{\mathrm{TP}+\mathrm{FP}}$$

(8) $$\mathrm{Recall}=\frac{\mathrm{TP}}{\mathrm{TP}+\mathrm{FN}}$$

TP is the true positive, FN is the false positive, and FP is the false positive of a model. 

The whole algorithm was run on a Macintosh with a 3 GHz processor and 16 GB memory based on the Python Keras Deep Learning Library.

## 3. Results

Table 1 shows the data used in our study, which was extracted from the filtered signal with 50% overlapping. During the annotation for the signal quality, a licensed technician went through the record first, and then picked out portions with cECG signals of consistent quality, after which the short segments were generated automatically for each portion.

**Figure 6:**
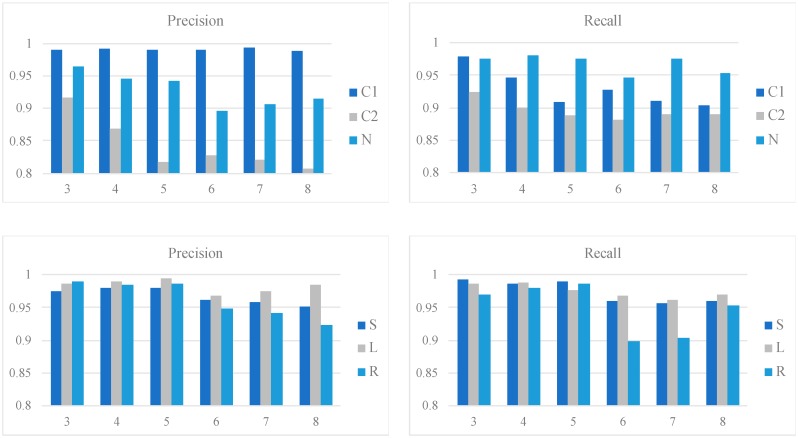
Class-wise performances of the qua_models and pos_models for the 2 s signal. The upper two bar plots show the precision and recall of the qua_models; whereas the lower two show those of the pos_models. The x-axis in each plot corresponds to the number of Conv layers.

**Figure 7:**
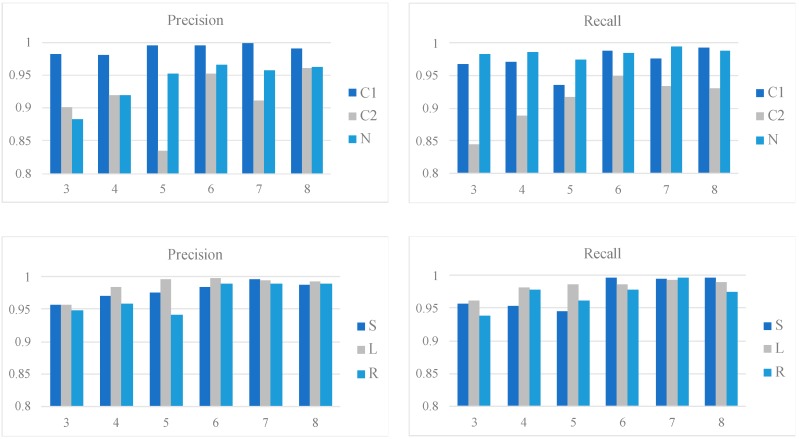
Class-wise performances of the qua_models and pos_models for the 4 s signal. The upper two bar plots show the precision and recall of the qua_models; whereas the lower two show those of the pos_models. The x-axis in each plot corresponds to the number of Conv layers.

Figure 6 shows the class-wise performances of the qua_models and the pos_models for the 2 s signal in terms of precision and recall; whereas Figure 7 shows the performances of models for the 4 s signal. In each bar plot of Figure 6 and Figure 7, the x-axis represents the number of the Conv layers. 

For the 2 s signal, the qua_model with three Conv layers outperforms the others given the results of C1 and C2. Specifically, the recall of C1 reaches a peak with three3 Conv layers. The pos_model with four Conv layers is the best in considering the averaged precision and recall combining the results of the three sleep positions. Specifically, the L can be detected more easily than the other two positions, given the higher recall and precision. On the other hand, the corresponding metrics are lower for S and R, which were mostly caused by the misclassification between S and R.

For the 4 s signal, the qua_model with six Conv layers is the best with a 0.99 precision and 0.99 recall for C1, which are slightly higher than the qua_model for 2 s signal. The pos_model reaches its best performance with a structure of seven Conv layers, with weighted averaged 0.99 precision and 0.98 recall. Compared to the best weighted averaged precision (0.98) and recall (0.98) of the 2 s pos_model, the pos_model for the 4 s signal with seven Conv layers is the best. These results suggest that the 4 s models are generally better than the counterparts for the 2 s signal.

Intriguingly, the S and R sleep positions were better classified in the 4 s model than in the 2 s model, making the precision and recall values for all three sleep positions very similar. Given that the 4 s pos_model requires more Conv layers than the 2 s pos_model (seven versus four) to reach the best performance, it may suggest a deeper network is advantageous in extracting the essential difference between the S and R positions. 

**Figure 8:**
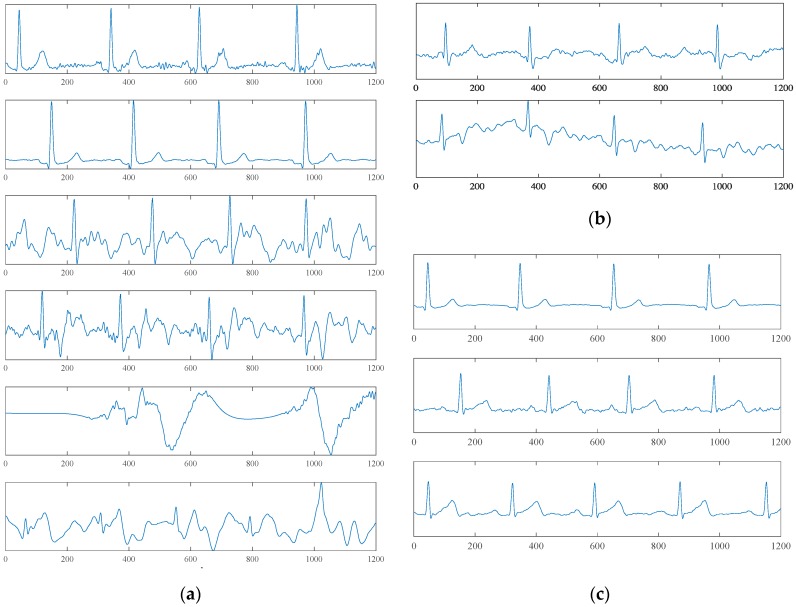
Exampling waveforms. (**a**) Exampling waveforms of 4 s time length correctly classified by the qua_model. The upper two subfigures belong to the C1 class, the middle two belong to the C2 class, and the lower two belong to the N class. (**b**) Misclassified samples of the C2 class. The upper sample was classified as C1; the lower sample was classified as N. (**c**) From top to bottom, the waveforms of the L, S, and R sleep positions.

These results suggest that the model can pick out the C1 signal accurately; on the other hand, the recall (0.98 in 4 s model of six Conv layers) of N class was also high enough to pick up most of the noisy samples. However, a portion of the C2 samples was misclassified as N. Figure 8b shows samples of the misclassified C2 by the qua_model. In view of the 0.95 precision of the C2 class, it suggests that even though some C2 samples were missed, the model can pick up the C2 precisely and it makes the following process, which would extract the physiological information, e.g., the heart rate (HR), simpler, since most of the detected C2 signals have prominent R peaks.

Noteworthily, this framework does not require R-peak detection and signal alignment, which is necessary for conventional ECG signal processing [12] (0.94 recall and 0.97 specificity in Reference [12]). If we compare the best qua_model with the preceding method by considering the C1 and C2 classes as one class that can be used to extract HR accurately, the weighted averaged recall and specificity are 0.98 and 0.94. If we only consider C1 as the useful signal, the recall and specificity are 0.99 and 0.99. 

The sample labeled as C1 by the qua_model was further input into the pos_model for sleep position classification. Comparing with the study of Takano et al. [10], the scheme for the position classification (supine and lateral in Reference [10]) is more specific in this study. Moreover, the sleep positions are better classified (supine: Recall = 0.89, precision = 0.87; lateral: Recall = 0.79, precision = 0.89 in Reference [10]).

## 4. Discussion

The unconstrained measurement of the cECG allows for practical use, and the long-term monitoring was justified by Barrett et al. [1]. Figure 8a shows that the hardware system can provide a clear ECG waveform, which could be used to extract meaningful heart-health information. However, the unconstrained measuring modality also causes critical issues in which information science must be incorporated to implement a fully automatic process so as to extract important physiological information without factitious intervention. This motivates us to construct a fully automatic classification framework without any predefined feature extractions or waveform alignments.

The dataset in this study consists of 12 subjects and it may seem small when we consider the practical use. We still think this framework is useful based on the following consideration. For personal home use, the personal cECG data will be accumulated quickly. What the framework needs in the initial stage are the models to classify the signal quality and sleep position accurately. The classified samples could be used to update the models using the transfer learning scheme so as to generate more robust models. Therefore, this framework can be used to discriminate the signal quality and sleep position in the initial stage. For a segment of 4-second length, the data amount of 30 min is 450. As indicated in Section 2.3, 10-fold CV that used 10% of the dataset as the independent test data were used to evaluate the models. For a dataset of 6000 samples, 600 samples (about 40 min) have been used to test the models. Judging from the results of the two models, they can classify the signal quality and sleep positions accurately for the initial practical use.

Overfitting is an unwanted phenomenon in a complicated machine learning model that a model learns from the training dataset perfectly and loses the generalization for new data. In deep learning, measures such as data augmentation, regularization, and early termination can be used to mitigate the overfitting problem. As it is explained in the methods section, dropout and data augmentation have been used. Moreover, models of different depth were constructed and tested using the 10-fold CV. The purpose of the comparison is to find the best structures of the two models, since overfitting can be confirmed with the divergence between the training accuracy and the test accuracy. From the results, it can be confirmed that neither too shallow (models with three or four convolutional layers) nor too deep (models with eight convolutional layers) reach the best performance, and these underperformances may be caused by underfitting and overfitting, respectively.

With regard to qua_model performance, the C2 class had the lowest recall. Figure 8b shows two C2 samples that were mistakenly labeled as C1 and N. The upper sample, which was classified as C1, has waveforms similar to C1 but high-frequency noise mixed in the signal. If we are going to use the cECG signal to analyze the ECG waveform in physiological/pathological reference, a successive discussion about the origin of these distorted waveforms is necessary. An additional signal that gives the mechanical information of the user, e.g., BCG, may be advantageous. The lower sample was classified as N. This misclassification may be caused by the higher amplitude of noise and baseline wandering. The low recall of C2 was mainly caused by the misclassification between N and C2, which caused the loss of a small portion of information of the signal. 

From the theory of the cECG (Figure 3), the L position has more prominent differences in the cECG waveform compared with the S and R positions. These theoretical differences were reflected in the results of pos_model for the 2 s signal. On the other hand, from Figure 8c, the difference between the S and R is visualized and may be perceived with a deeper network. As we have seen in the results, by a deeper network for a longer 4 s signal, the S and R can be classified more accurately. Unlike Takano’s study, which tried to separate the sitting, supine, and lateral positions using multimodal signals that included cECG [10], we focused on using the intrinsic properties of cECG to implement a more detailed classification for sleeping positions. By using the CNN-based model, the class-wise precisions and recalls were improved significantly. 

Our framework relies on a pre-trained model to implement the classification, and the time required to compute the prediction of quality/corresponding sleep position of a new sample is less than 1 ms. Therefore, this framework can be applied to online monitoring.

In view of the intrinsic differences among users, e.g., the anatomic difference of the heart, we need to be careful in applying the trained model to a new user. On the other hand, in considering the results, it is obvious that the models can learn from the training data regarding the signal quality and sleep positions to make accurate predictions. This availability of the framework is the main concern of this study. To confirm the reproducibility of the models, we asked one subject to repeat the measurement and tested the trained models with the new samples (S: 235, L: 105, R: 108). The precisions of the trained pos_model for S, L, and R positions are 1.00, 0.90, 1.00; whereas the recalls are 0.98, 0.97 and 0.98. We believe that with appropriate training dataset, the framework can be used to construct accurate models for a new user.

This study shows the results of harnessing unconstrained cECG signals with the CNN techniques for practical use of this unconstrained sensing technique. The results of this framework can be viewed as a potential solution of preprocessing framework for a simplified condition, where all the samples were assumed as normal and we did not need to take the arrhythmias into consideration. If the cECG system is applied in diagnosis assistance, some critical issues should be addressed.

As we see from the samples belonging to the N class in Figure 8a, different waveform patterns exist in this class and the noise could be influenced by different factors, more or less, such as sleep position change or limb movement. Noise signals caused by different external factors should have different patterns, therefore, it may be more appropriate to use the unsupervised scheme to learn the whole sampling space of the cECG first. For example, a clustering approach that is based on distance metrics and for the latent feature generation [20] may be advantageous. Moreover, some dangerous ventricular arrhythmias such as the Torsades de pointes (TDP) are similar to the noise signal, and the framework should detect these pathological signals from the noise.

Moreover, the CNN-based models could reach satisfactory accuracies for the simplified condition in this study based on the waveform of a cECG signal, however, it cannot distinguish the origins of the distorted waveform. Looking at the waveforms in the bottom of Figure 8a, it is, more or less, similar to the waveform of TDP. An additional signal that can provide information about the user from another angle about the origin of the distorted waveform, e.g., the BCG, would be indispensable in this problem. 

Finally, the authors here advocate a paradigm shift for the signal processing and modeling for the wearable/unconstrained devices. Since the unstable noise is an unavoidable characteristic and given their feature of continuous measurement, an approach that can work well with the noise is necessary. In other words, the noise should not be excluded in the approach but should be taken into account as one or multiple classes with different patterns. To this end, the method based on predefined features extraction may not be the solution anymore. Instead, the algorithms that can generate the latent features in accordance with the specific problem should be a better choice. 

## 5. Conclusions

The cECG has promising applications to personal healthcare in its unconstrained and ready-to-use properties. To make full use of the electrical signal, a fully automatic preprocessing framework that can work with signals whose quality may change abruptly and a waveform that varies more conspicuously than a clinical ECG is indispensable. This study proposed a framework consisting of a signal quality classifier (qua_model) and a sleep position classifier (pos_model) using CNN techniques that learn the latent features for specific problems via backward propagation. 

By dividing the signal quality into three classes, the qua_model can recognize short signal segments with averaged precision (0.97) and sensitivity (0.97) over 10-fold cross-validation. The clear cECG signal was input into the pos_model for sleep position classification. The precision and recall for supine, left, and right lateral position are: Precision = (0.99, 0.99, and 0.99) and recall = (0.99, 0.99, 0.99), respectively. 

This study proposes a prepressing framework that requires little signal manipulation, such as fiducial point detection and alignment, and has improved the classification outcome for the sleep position. Therefore, this study may serve as an important step trying to explore the cECG technique for unconstrained heart monitoring.

## Figures and Tables

**Table 1:** Description of the data used in training and model evaluations.

	Model	Pos. ^1^					
		2s			4s		
Data		L	R	S	L	R	S
**Qua. ^1^**	C1	1567	2397	2498	928	1144	1903
	C2	1175	678	343	583	335	170
	N	1930	147	1392	845	73	569
Position-wise aggregation		4672	3222	4233	2356	1552	2642

^1^ “Qua.” denotes the qua_model; “Pos.” denotes the pos_model.
